# CFAssay: statistical analysis of the colony formation assay

**DOI:** 10.1186/s13014-015-0529-y

**Published:** 2015-11-04

**Authors:** Herbert Braselmann, Agata Michna, Julia Heß, Kristian Unger

**Affiliations:** Research Unit Radiation Cytogenetics, Helmholtz Zentrum München, German Research Center for Environmental Health GmbH, Neuherberg, Germany; Clinical Cooperation Group ’Personalized Radiotherapy of Head and Neck Cancer’, Helmholtz Zentrum München, German Research Center for Environmental Health GmbH, Neuherberg, Germany

**Keywords:** Colony formation assay, Cell survival, Linear-quadratic model

## Abstract

**Background:**

Colony formation assay is the gold standard to determine cell reproductive death after treatment with ionizing radiation, applied for different cell lines or in combination with other treatment modalities. Associated linear-quadratic cell survival curves can be calculated with different methods. For easy code exchange and methodological standardisation among collaborating laboratories a software package CFAssay for R (R Core Team, R: A Language and Environment for Statistical Computing, 2014) was established to perform thorough statistical analysis of linear-quadratic cell survival curves after treatment with ionizing radiation and of two-way designs of experiments with chemical treatments only.

**Methods:**

CFAssay offers maximum likelihood and related methods by default and the least squares or weighted least squares method can be optionally chosen. A test for comparision of cell survival curves and an ANOVA test for experimental two-way designs are provided.

**Results:**

For the two presented examples estimated parameters do not differ much between maximum-likelihood and least squares. However the dispersion parameter of the quasi-likelihood method is much more sensitive for statistical variation in the data than the multiple *R*^2^ coefficient of determination from the least squares method.

**Conclusion:**

The dispersion parameter for goodness of fit and different plot functions in CFAssay help to evaluate experimental data quality. As open source software interlaboratory code sharing between users is facilitated.

**Availability:**

The package is available at http://www.bioconductor.org/packages/release/bioc/html/CFAssay.html.

## Background

Clonogenic assay or colony formation assay (CFA) is an *in vitro* cell survival assay based on the ability of single cells to grow into colonies [1]. It is the gold standard to determine cell reproductive death after treatment with ionizing radiation. Whereby the relationship between the radiation doses and the proportion of surviving colonies is usually described by parametric cell survival curves. These can be used for the characterisation of the radiation sensitivity of different tumour cell lines given a specific radiation type [2], or in combination with other treatment modalities, e.g. a therapeutic agent or radiation sensitizer [3]. For the analysis of cell survival curves CFAssay uses the commonly used linear-quadratic model (LQ model) [1]. Apart from radiation the CFA is also applicable to two-way experimental designs, in which typically a control cell line and a genetically modified cell line are treated with a therapeutic drug [4]. In CFAssay ANOVA based tests are used for two-way designs.

Usually, simple least square (LS) methods are applied on the logarithmic survival fractions in order to calculate parameter values for the LQ model and to perform comparison tests between curves or between mean values of survival fractions. The statistical analysis with the LS method requires that the data can be described accurately with the normal distribution. However, because colony numbers are discrete values following the Poisson distribution between different cell culture plates of a particular experiment, maximum likelihood (ML) based methods are preferable from a statistical point of view. The ML approach for cell survival curves was introduced by [5] using a one-hit-multi-target model. Assuming that the model (here LQ) is applicable, ML estimations of the model parameters become asymptotically efficient, i.e. are most precise (for practical and theoretical considerations in general see for e.g. [6]). The efficiency can be demonstrated in a simplified mean value example: For two numbers *k*_1_ and *k*_2_ of surviving colonies in plates with *N*_1_ or *N*_2_ cells seeded ML yields the pooled mean *m*_1_=(*k*_1_+*k*_2_)/(*N*_1_+*N*_2_) and LS yields the arithmetic mean *m*_2_=(*k*_1_/*N*_1_+*k*_2_/*N*_2_)/2. When *μ* is the expectation of *m*_1_ and *m*_2_, the Poisson variance of *m*_1_ is *μ*/(*N*_1_+*N*_2_) and that of *m*_2_ is (*μ*/*N*_1_+*μ*/*N*_2_)/4. The relative efficiency of *m*_2_ to *m*_1_ is the ratio of the two variances, 4/(1/*N*_1_+1/*N*_2_)/(*N*_1_+*N*_2_). For equal cell numbers the relative efficiency is 1.0 and in addition *m*_1_=*m*_2_. When the cell numbers are different by a factor of 3, the relative efficiency is 0.75.

In CFAssay the ML method is set as default but for the sake of comparison the LS method can be optionally chosen. The ML method provides two related quantities, the so called deviance and a dispersion parameter, which are useful to assess the quality of the data or the goodness of fit. Both quantities are more sensitive against outliers than the coefficient of determination *R*^2^ in LS regression. The dispersion parameter is derived from the method of iteratively reweighted least squares which solves the ML equations when they can be formulated as a generalized linear model [7]. This holds true for the LQ model in the CFA as well as for the comparison of mean survival fractions with help of ANOVA models. Generalized linear models or LS regression are processed by the R-functions glm or lm, respectively. The functions of CFAssay serve as wrappers of these functions to simplify its use for the analyst and to extract numerical results along with the terminology used for the CFA. In addition to the LS or ML method, an option for the weighting of the LS as described in [1] is provided. For visual inspection of data quality a further function is provided for plotting cell survival curves for each replicated experiment, annotated with the value of its contribution to the total weighted residual sum of squares. The package can be installed directly in R using the commands source(“http://bioconductor.org/biocLite.R,”) and biocLite(~CFAssay~). Once installed the reference manual can be accessed from R using the command browseVignettes("CFAssay").

## Methods

After any irradiation with a dose *d* the number of scored colonies *y* is proportional to *N*, the number of cells seeded and to the average proportion *S*=*S*(*d*) of cells that grow into colonies. Thus, the Poisson probability for *y* is given by

(1) $$ prob(y)=e^{-NS}(NS)^{y}/y!  $$

### The linear-quadratic cell survival model

Dose dependent surviving fractions in CFAssay are fitted by the LQ-model

(2) $$ S = S(d) = e^{-c-\alpha d-\beta d^{2}}  $$

where *d* is the radiation dose measured in Gy or another unit, *α* is the dose effect per Gy and *β* per Gy^2^. *c*=−*log*(*S*(0)), represents the logarithmic plating efficiency, i.e. the surviving fraction of unirradiated cells, which varies between different experiments. Usually, when taken as a fixed value, the plating efficiency is put by division on the left side of the equation^1^. We leave it on the right side to have the possibility to fit it together with the other two parameters. Statistically, colonies from untreated cells are as well as colonies from treated cells random observations.

### ANOVA model for the two-way experiment

For the analysis of the two-way experiment we use multiplicative modelling, i.e. a logarithmic linear (log-linear) model with two linear factors *A* and *B* and a factor *D* for their potential interaction. Then the model can be formulated as

(3) $$ S = e^{c+Ax_{1}+Bx_{2}+Dx_{1}x_{2}}  $$

or as nested parametrization

(4) $$ S = e^{c+Ax_{1}+B_{0}x_{2}+(B_{1}-B_{0})x_{1}x_{2}}  $$

where *A*, *B* are the effects of cell line modification and of one or of two different treatments, *D* the interaction effect and *x*_1_, *x*_2_ are 0 or 1, dependent on which factor is applied. Thus, interaction for applied *A* and *B* means that there is more (or less) effect than the sum. In the second, nested parametrization *B*_0_ is the effect of treatment in control cells (*x*_1_=0) and *B*_1_ the treatment effect after genetic modification of the cell line (*x*_1_=1), for e.g. siRNA knockdown of a gene of interest. The interaction *D* is then the difference between *B*_0_ and *B*_1_. *c* represents the logarithmic plating efficiencies in replicated experiments, similar as in the LQ model (2).

Finally, with the ML method the model parameters are determined such that the joint probability according to (1) for the set of all colony counts *y*_*ij*_ at all doses *d*_*i*_ (or treatments) and for all replicates *j* is maximized. For the Poisson distribution this is equivalent to iteratively minimize the sum of weighted squared differences between observed (*S*_*ij*_=*y*_*ij*_/*N*_*ij*_) and modeled survival fractions *S*, with inverse Poisson variances as weights. For overall Poisson distribution, the dispersion parameter, defined as the sum of weighted least squares divided by its expected value ([6]), should be about 1.0. However, for the CFA it often appears to be >1.0, mainly due to extra variability between replicated experiments, even after correction for plating efficiencies. Therefore the calculated standard deviations in CFAssay are scaled by the square root of the dispersion parameter. This technique is also called quasi-likelihood and uses the quasipoisson family of the R-function glm. With the LS method simply the sum of squared differences between observed (*log*(*S*_*ij*_)=*log*(*y*_*ij*_/*N*_*ij*_)) and modeled logarithmic survival fractions *log*(*S*) according to (2)–(4) is minimized. For assessment of the goodness-of-fit of the LS method the multiple *R*^2^ (coefficient of determination) is calculated, which describes the fraction of variability in the total data which can be explained by model dependency and plating efficiencies.

### Example data

We demonstrate the ML method for two examples. For cell survival curves it is demonstrated on colony counts of irradiation experiments with a pair of two human head and neck squamous cell carcinoma (HNSCC) cell lines, CAL33 [8] and OKF6/TERT1 [9] which were irradiated with five different doses up to 6 Gy. The second example was taken from [4]. There, the treatment effect of one given dose of the chemotherapeutic drug cisplatin/5-FU was tested for the human oesophageal adenocarcinoma cell line OE19 before and after COX7A2 knockdown by siRNA transfection. It was shown that knockdown of the COX7A2 protein altered chemosensitivity, which appeared statistically as an interaction effect. Data of the two examples are supplied in the CFAssay package.

## Results

### Cell survival curves

First, with the CFAssay function cellsurvLQfit we fit the LQ model to colony counts of the OKF6/TERT1 cell line. R commands for the assessment of results are shown in Table 1. The ML method yields *α*=0.52±0.06/Gy, *β*=0.021±0.010/Gy^2^ with a dispersion parameter 4.34 which is significantly greater than one (*χ*^2^-test, d.f. = 38, *p*<0.05). In spite of statistical significance, a critical limit for the dispersion parameter depends on experience and may vary between different labs. A value of 9.0 corresponding to 3 Poisson standard deviations might be a recommendation in order to take a closer look for outlying points or experiments for potential removal or replacement. In comparison the LS methods yields *α*=0.54±0.07/Gy, *β*=0.023±0.011/*Gy*^2^ with a residual square sum of 3.35 and a coefficient of determination *R*^2^=0.99. For the results the plating efficiencies were fitted together with the data from irradiated samples. Fixed plating efficiencies, derived by option PEmethod = “fix” in the function cellsurvLQfit result in almost identical coefficients but the dispersion parameter of the ML method becomes 9.73. This is just an effect of shift on the logarithmic scale, because the shape of the mean curve gets larger distance to the single replicated experiments for treated samples when forced to pass the observation at dose zero.

**Table 1:** R commands for the two presented examples

R Command	Comment
LQ cell survival curves	
> library(CFAssay)	Loads the package
> filename <- ~expl1_cellsurvcurves.txt~	
> datapath <- system.file(~doc~, filename, package=~CFAssay~)	Gets the path to the example data
> datatab <- read.table(datapath, header=TRUE, sep=~\t~)	Reads the data
> X <- subset(datatab, cline==~okf6TERT1~)	Selects OKF6 cell survival data
> print(cellsurvLQfit(X, method=~ml~))	Fits LQ model with maximum likelihood
> print(cellsurvLQfit(X, method=~ls~))	Fits LQ model with least squares
> print(cellsurvLQdiff(datatab, curvevar=~cline~))	Compares curves from two cell lines
Knockdown and treatment experiment	
> filename <- ~exp2_2waycfa.txt~	
> datapath <- system.file(~doc~, filename, package=~CFAssay~)	Gets the path to the example data
> datatab <- read.table(datapath, header=TRUE, sep=~\t~)	Reads the data
> print(cfa2way(datatab, A=~siRNA~, B=~x5fuCis~, param=~A/B~, method=~ml~))	Fits LQ model with maximum likelihood
> print(cfa2way(datatab, A=~siRNA~, B=~x5fuCis~, param=~A/B~, method=~ls~))	Fits LQ model with least squares

**Fig. 1:**
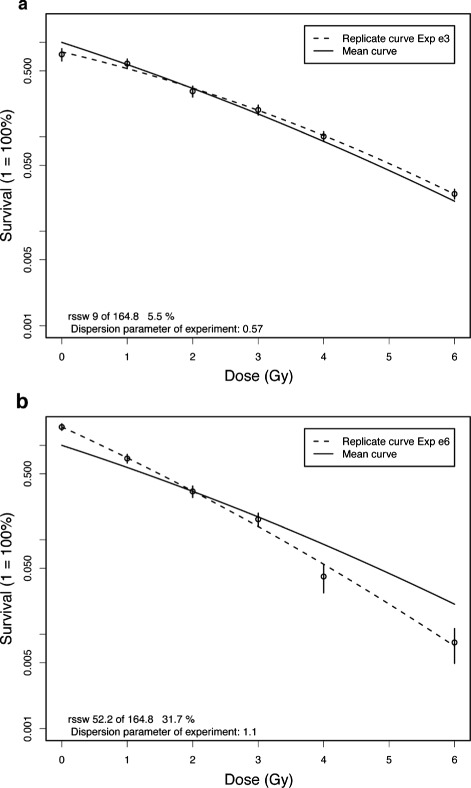
Diagnostic plots of linear-quadratic cell survival curve (OKF6/TERT1) fitted by maximum-likelihood. Solid curve: mean of 8 replicate experiments, dashed curves: 2 of 8 experiments. Annotated is the percentage of the residual sum of weighted squares to total 164.8 **a**: 5.5 %, **b**: 31.7 %, expected: 12.5 %

Diagnostic plots of the mean curve versus curves from single replicates are shown for two experiments in Fig. 1a and b. One of these experiments contributes more than 30 % to the residual weighted sum of squares. The within experiment fit is good (dispersion parameter 1.1) but the slope is stronger than that of the mean curve. When we include artificially introduced overdispersion into the data, for e.g. by changing the number of colonies for one measurement, so that the dispersion parameter becomes larger than 8.0, then the *R*^2^ decreases only to 0.97. Thus, the dispersion parameter for the ML method is more sensitive against outlying points or outlying experiments and thereby provides a better quantity for the diagnostic assessment of the experimental results. With the function cellsurvLQdiff the OKF6/TERT1 cell line is compared with the CAL33 cell line using the ANOVA F-test, which is the preferred test for generalized linear models in the presence of overdispersion. For this test one LQ curve is fitted to the total cell survival data (model 1) and in contrast two LQ curves are fitted separately to the cell lines (model 2). The p-value is the probability that the difference between the residual data scatter of model 1 compared to that of model 2 occurs by chance. For this example both methods indicate a significant overall difference (*p*=0.0015 with ML, *p*=0.0006 with LS).

### Knockdown and treatment experiment

**Fig. 2:**
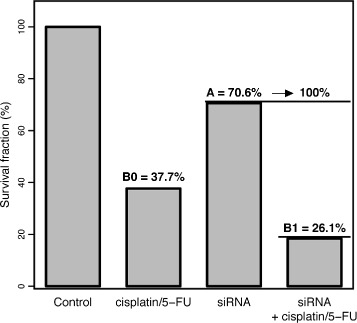
Influence of siRNA transfection for COX7A2 on sensitivity for cisplatin/5-FU. The height of the bars represent cell survival fractions relative to the control sample. Annotated are values as calculated in the two-way ANOVA according to Eq. (4), converted to percentages. The value of B1 corresponds the height of the fourth bar relative to the third bar. The difference between B1 and B0 is significant (ML method, F-test, *p*=0.012)

By the experimental design four groups were defined: control cells, treated control cells, knockdown cells and treated knockdown cells. The experiment was replicated 4-times and the influence of the two factors *knockdown* and *treatment* was analysed by model Eq. (4). We set *A* the effect of COX7A2 knockdown on survival reduction, *B*_0_ the effect of treatment in control cells and *B*_1_ the treatment effect after knockdown. The results are illustrated in Fig. 2. The resulting values were *A*=−0.348±0.053 (70.6 %), *B*_0_=−0.976±0.072 (37.7 %) and *B*_1_=−1.343±0.095 (26.1 %). Further, the F-test indicated significant interaction (*p*=0.012). The dispersion parameter was 4.15 (d.f. = 9, *p*<0.05) indicating some extra variation compared to the Poisson variance. The diagnostic plots of CFAssay (not shown) show somewhat larger deviation from the expected mean values for one of the 4 experiments. The least squares method based on the logarithmic survival fractions yielded similar results (*A*=−0.311±0.092, *B*_0_=−0.975±0.092, *B*_1_=−1.342±0.092, F-test *p*=0.019). However the coefficient of determination is *R*^2^=0.996 which indicates a good fit because it is not sensitive against deviations based on the Poisson variance.

## Discussion

We established the software package CFAssay for statistical analysis of the colony formation assay and to be used with the open source statistic software R [10]. The package consists of several functions for the calculation of linear-quadratic (LQ) cell survival curve parameters, plotting of survival curves and a statistical test for comparing pairs of survival curves. In addition, it contains a function for ANOVA testing of two-way experimental designs with the CFA. The functions use per default maximum likelihood (ML) based methods, however optionally the least square (LS) method or a weighted LS method with weights calculated according to [1] can be used for sake of comparison. Results of the ML method are known to be most stable when the data vary according to the Poisson distribution and the model can be assumed to be appropriate. Data of the CFA is usually analysed by the LQ model [3]. Although, as in the presented examples, where numbers of survived colonies are throughout two-digit (>10) or more, ML and LS lead to comparable results, this cannot be guaranteed in general. However, with the ML method the dispersion parameter provides a sensitive quantity to assess the quality of the data. Large dispersion values can be due to outlying single points of one experiment or to variation between experimental replicates. Deviations from the LQ model should not have a substantial statistical influence for irradation doses below 8 Gy. In the manual we recommend roughly a critical dispersion value of 9.0 in analogy to the three-sigma rule. However, it depends on experience and CFAssay provides diagnostic plots for single experiments.

The LQ model for cell survival and log-linear ANOVA for Poisson distributed counts of surviving colonies belong statistically to a wider class of so called generalized linear models [7]. Numerical procedures for its solution with the algorithm of iteratively reweighted least squares, which solve the ML equations, are now available with almost every software for statistical analysis (GENMOD in SAS [11], GENLIN in SPSS [12], glm in R).

## Conclusions

The availability of numerical procedures for the ML method and its features for thorough statistical analysis are a reason why it should be taken into account. R is now the most widely used statistical framework for the professional statistician and also non-statisticians such as biologists. Because it is free-available for everyone, interlaboratory code sharing between users is facilitated. CFAssay significantly simplifies the use of the R functions glm and lm for non-statisticians working with the CFA and allows straightforward analysis and plotting of CFA data. The package is open for extensions to other models for cell survival and related statistical analysis.

## Endnote

^1^ The survival fraction SF is then defined as *S*(*d*)/*S*(0).

## Abbreviations

CFA: Colony formation assay
LQ model: Linear-quadratic model
ANOVA: Analysis of Variance
LS: Least squares
ML: Maximum likelihood
Gy: Gray
